# Molecular Brushes with a Polyimide Backbone and Poly(ε-Caprolactone) Side Chains by the Combination of ATRP, ROP, and CuAAC

**DOI:** 10.3390/polym13193312

**Published:** 2021-09-28

**Authors:** Anna V. Kashina, Tamara K. Meleshko, Natalia N. Bogorad, Viktor K. Lavrentyev, Alexander V. Yakimansky

**Affiliations:** Institute of Macromolecular Compounds, Russian Academy of Sciences, Bolshoy pr. 31, 199004 Saint Petersburg, Russia; meleshko@hq.macro.ru (T.K.M.); bogorad@hq.macro.ru (N.N.B.); lavrentev1949@mail.ru (V.K.L.); yakimansky@yahoo.com (A.V.Y.)

**Keywords:** molecular brushes, polyimide, poly(ε-caprolactone), ATRP, ROP, CuAAC

## Abstract

An approach to the synthesis of the novel molecular brushes with a polyimide (PI) backbone and poly(ε-caprolactone) (PCL) side chains was developed. To obtain such copolymers, a combination of various synthesis methods was used, including polycondensation, atom transfer radical polymerization (ATRP), ring opening polymerization (ROP), and Cu (I)-catalyzed azide-alkyne Huisgen cycloaddition (CuAAC). ATRP of 2-hydroxyethyl methacrylate (HEMA) on PI macroinitiator followed by ROP of ε-caprolactone (CL) provided a “brush on brush” structure PI-*g*-(PHEMA-*g*-PCL). For the synthesis of PI-*g*-PCL two synthetic routes combining ROP and CuAAC were compared: (1) polymer-analogous transformations of a multicenter PI macroinitiator with an initiating hydroxyl group separated from the main chain by a triazole ring followed by ROP of CL, or (2) a separate synthesis of macromonomers with the desirable functional groups (polyimide with azide groups and PCL with terminal alkyne groups), followed by a click reaction. Results showed that the first approach allows to obtain graft copolymers with a PI backbone and relatively short PCL side chains. While the implementation of the second approach leads to a more significant increase in the molecular weight, but unreacted linear PCL remains in the system. Obtained macroinitiators and copolymers were characterized using ^1^H NMR and IR spectroscopy, their molecular weight characteristics were determined by SEC with triple detection. TGA and DSC were used to determine their thermal properties. X-ray scattering data showed that the introduction of a polyimide block into the polycaprolactone matrix did not change the degree of crystallinity of PCL.

## 1. Introduction

Polyimides (PI) are an essential class of polymers with a set of valuable properties. In addition to heat, thermal, radiation, and chemical resistance, good film-forming properties, high mechanical strength, and excellent insulating properties, polyimides demonstrate good biocompatibility in vitro and in vivo, as well as low toxicity [1,2,3]. Multicomponent copolymers, in which PI blocks are combined with blocks of biocompatible aliphatic polymers, have great potential for the development of materials for tissue engineering.

Although atom transfer radical polymerization (ATRP) has been successfully used to synthesize many types of copolymers, it remains a challenge to synthesize copolymers of different architectures using monomers that polymerize through fundamentally different mechanisms, such as ATRP and ring opening polymerization (ROP). To solve this problem, one can use initiators containing two types of functional groups capable of initiating each of these processes in parallel and independently [4,5]. Second approach is introduction of a functional group that initiates the polymerization of the second monomer at the stage of initiation or termination of the first monomer polymerization (post-modification) [6]. 

Thus, in [7], graft copolymers with poly(2-hydroxyethyl methacrylate) (PHEMA) backbone and block copolymer side chains containing blocks of poly(ε-caprolactone) PCL and poly(butyl acrylate) (PBA) were synthesized. Copolymers of PCL with poly(octadecyl methacrylate) (POMA) and poly(N,N-dimethylamino-2-ethyl methacrylate) (PDMAEMA) PCL-*b*-PODMA-*b*-PDMAEMA or PCL-*b*-(PODMA-*co*-PDMAEMA) were obtained in [5], in which the PODMA block had a "pseudo-brush" structure due to long aliphatic tails. Another example of a successful combination of the different methods of controlled polymerization is the synthesis of a three-armed star-shaped polymer with arms of different structures, for which different methods were used [4]. Thus, the PCL arm was obtained using ROP, the polystyrene (PS) arm was obtained by the NMP, and the poly(tert-butyl acrylate) (PTBA) was obtained by the ATRP.

The ROP is effectively combined with other CRP methods in the synthesis of various copolymers. Thus, in [8], the authors describe the preparation of triblock copolymers with a central PCL block and peripheral PDMAEMA blocks using a combination of ROP with RAFT. Synthesized by ROP PCL with hydroxyl end groups was functionalized using 4-cyanopentanoic acid dithionaphthalenoate (CPADN) and used as a macro-RAFT agent for polymerization of DMAEMA to obtain the targeted triblock copolymers. An efficient method for the synthesis of linear di- and triblock copolymers with PCL blocks with narrow molecular weight distribution is to carry out ROP on mono- or difunctional polymer initiators. ROP of CL on the corresponding polymer initiators with hydroxyl chain ends provides copolymers in which PCL blocks are covalently attached to a block of polydimethylsiloxane [9], polyisobutylene [10], and poly(ethylene oxide) [11].

Grafting of polyester chains with narrow molecular weight distribution, in particular, PCL for the production of molecular brushes is also of great interest. The method of click chemistry is often used to graft PCL chains [12,13]. For example, in [12], the surface modification of the nanodispersed cellulose systems by grafting PCL chains using click-chemistry is described. However, to obtain molecular brushes with polyester side chains, ROP is easier to perform [14]. Therefore, numerous articles are devoted to the synthesis of molecular brushes with homopolymer PCL side chains or block copolymer side chains with a PCL block [12,13]. Carrying out polymerization of CL on multifunctional polymer initiators with varying numbers and positions of initiating groups leads to the production of PCL blocks with narrow molecular weight distribution, which are usually grafted to the carbochain backbone. To our knowledge, there are no data on the introduction of PCL blocks into the side chains of molecular brushes with a polyarylene backbone.

At the same time, works on combining aromatic PIs with aliphatic polyesters, in particular with PCL, have been going on for more than two decades. With simple mixing of homopolymer PI and PCL, for example, through a common solvent, phase separation of polymers is observed at the macroscopic level [15]. A more uniform phase system of PI and PCL is obtained by synthesizing chemically bonded PI and PCL blocks [15,16,17,18,19,20]. Until recently, the covalent bonding of these polymers was carried out either by stepwise polycondensation, leading to the production of linear alternative segmented (or multisegmented) block copolymers [18,19], or by cross-linking between PI and PCL chains in a polymer mixture or between PI and PCL layers in a multilayer coating [16,17,20]. A valuable feature of such copolymers is microphase separation [17], which gives them new interesting, for example, membrane properties. Thus, based on multisegmented linear block copolymers containing segments of polyurethanimide and PCL, effective diffusion membranes have been developed for the separation of binary mixtures of organic liquids [17]. However, the preparation of such segmented block copolymers is very laborious. Another method for combining PI and PCL by preparing block copolymers with sequentially attached PI and PCL blocks was proposed in [21]. In [22] the synthesis of linear block copolyimide with PCL and PI blocks based on 3,3’-dioxybenzidine was proposed using ROP of CL on a difunctional polyimide initiator. The obtained copolyimide was intended to improve the dispersion of carbon nanotubes in low-boiling organic solvents.

It should be noted that the introduction of PCL blocks into multiblock copolymers is of considerable interest from the point of view of further applications of these copolymers, since PCL blocks are capable of undergoing alkaline [23] and plasma [24] etching and biodegradation. The synthesis of new initiators and ROP of monomers and macromonomers containing functional groups on such initiators is a promising strategy for the preparation of macromolecules of complex architecture. In recent years, due to the development of the ROP method, it has been successfully used to obtain copolymers of different chemical structures and architecture.

Earlier, our research group reported the synthesis of triblock copolymers based on polyimide with external blocks of poly(ε-caprolactone) (PCL) [25], as well as grafted pentablock copolymers of linear-brush topology with external PCL and PMMA blocks and an internal brush-type block PI-*g*-PMMA [26]. This work is devoted to the development of methods for the synthesis of previously undescribed molecular brushes with a PI backbone and PCL side chains. To obtain such copolymers, a combination of various synthesis methods was used, including polycondensation, ATRP, ROP, and Cu (I) catalyzed azide-alkyne Huisgen cycloaddition (CuAAC) [27].

## 2. Materials and Methods

### 2.1. Materials

N-methyl-2-pyrrolidone (N-MP, 98%, Aldrich, St. Louis, MO, USA), toluene (analytical grade) methylene chloride, chloroform, THF, DMF (all reagent grade, Vekton, Voronezh, Russia), trimethylamine (≥99%, Aldrich, Overijse, Belgium), and pyridine (99%, Acros Organics, NJ, USA) were purified through standard techniques. 3,3′,4,4′-(1,3-diphenoxybenzene)tetracarboxylic dianhydride (99%, Ambinter Stock Screening Collections, China), 2,4-diaminophenol dihydrochloride (98%, Lancaster, Eastgate, White Lund, Morecambe, England), and potassium iodide (≥99.5%, Aldrich, St. Louis, MO, USA) were dried at elevated temperature in a vacuum prior to synthesis. 2-Hydroxyethyl methacrylate (HEMA) (99%, Acros Organics, UK) and ε-caprolactone (99%, Aldrich, St. Louis, MO, USA) were distilled twice under vacuum. Copper(I) chloride (99%, Aldrich, St. Louis, MO, USA) was stirred in glacial acetic acid overnight, filtered, and washed with argon-purged methanol (reagent grade, Vekton, Russia). Sn(II) 2-ethyl hexanoate (Sn(EH)_2_) (~95%, Aldrich, Japan) was distilled under vacuum. 2-Bromoisobutyryl bromide (98%, Aldrich, St. Louis, MO, USA), 2,2’-bipyridine (bpy) (≥99%, Aldrich, St. Louis, MO, USA), sodium azide (NaN_3_) (≥99.5%, Aldrich, St. Louis, MO, USA), propargyl alcohol (99%, Aldrich, Steinheim, Germany), N,N,N′,N′′,N′′-pentamethyldiethylenetriamine (PMDETA) (99%, Aldrich, Steinheim, Germany), copper(I) bromide (CuBr) (98%, Aldrich, St. Louis, MO, USA), and LiBr (≥99%, Aldrich, St. Louis, MO, USA) were used without additional purification.

### 2.2. Synthesis of PI-g-PHEMA as Branched Multicenter Macroinitiator with Hydroxyl Groups in Each Repeating Unit of the Side Chains of the Grafted Copolyimide

**Figure 1:**
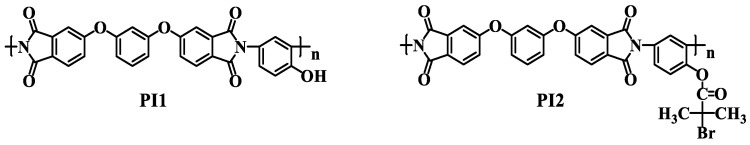
Multifunctional polyimide macroinitiators.

The polyimide multifunctional macroinitiator with initiating groups in almost every monomer unit was obtained by polycondensation of dianhydride of 3,3′,4,4′-(1,3-diphenoxybenzene)-tetracarboxylic acid and 2,4-diaminophenol, its phenol groups being further modified by 2-bromoisobutyryl bromide. The initial PI1 and the related PI2 multifunctional macroinitiator (Figure 1, see Result and Discussion section) were synthesized using procedures described in our previous publications [28,29,30].

To obtain a brush-type macroinitiator with hydroxyl groups in the side chains, graft copolyimide PI-*g*-PHEMA was synthesized by polymerization of 2-hydroxyethyl methacrylate (HEMA) on PI2 macroinitiator (Figure 1) by the ATRP. A typical synthesis procedure was as follows. A weighed portion of PI2 (0.075 g, 0.11 mmol per initiating group) and 2,2′-bipyridine (0.052 g, 0.33 mmol) was placed into a 25 mL Schlenk flask equipped with a magnetic stirrer, then DMF (16.5 mL, 0.21 mol) and HEMA (3.65 mL, 0.03 mol) were added with the syringe. The solvent, monomer, and syringes were purged with argon. The flask was sealed with a rubber septum, and the mixture was stirred until the powder was completely dissolved. Then, three freeze−pump−thaw cycles were carried out (evacuation for 15 min), after which the flask was filled with argon. After opening the septum in a stream of argon, CuCl (0.011 g, 0.11 mmol) was added to the reaction mixture, after which the flask was closed again with the septum. Three more freeze−pump−thaw cycles (evacuation for 15 min) of the reaction mixture were carried out. The flask was filled with argon and thermostated in an oil bath, and placed on a magnetic stirrer with a temperature controller, at 30 °C.

After a given reaction time, the reaction mixture was quickly cooled to room temperature and diluted two times with THF. To remove copper salts from the mixture, reaction solution was passed through a column filled with Al_2_O_3_, then concentrated using a rotary evaporator, and the polymerization product was precipitated into a water/methanol mixture with a volume ratio of 1/6. The filtered powder was dried under vacuum at 50 °C.

### 2.3. Synthesis of Graft Copolymers PI-g-(PHEMA-g-PCL)

The synthesis of the copolymer was carried out by ROP of CL in bulk on PI-*g*-PHEMA. A typical synthesis procedure is as follows: 0.05 g (0.23 mmol per initiating group) of PI-*g*-PHEMA was added to a 25-mL Schlenk flask equipped with a magnetic stirrer, and three freeze−pump−thaw cycles were carried out (evacuation for 15 min), after which the flask was filled with argon. Then, 1.2 mL (0.011 mol) of ε-caprolactone was introduced in an argon flow and thermostated at 130 °C using an oil bath. After complete dissolution of the macroinitiator, 0.05 mL (0.15 mmol) of Sn(Oct)_2_ was introduced into the reaction in an argon flow. Upon completion of polymerization, the reaction mixture was cooled to room temperature and diluted with methylene chloride. The resulting solution was passed through a silica gel column to purify the product from catalyst and monomer impurities. The solution was then concentrated on a rotary evaporator and the product was precipitated into cooled petroleum ether. The polymer was dried under vacuum at 30 °C.

### 2.4. Introduction of Azide Groups into Polyimide 

The functionalization of the polyimide with azide groups was carried out as follows. A weighed portion of PI2 macroinitiator (0.5 g, 0.66 mmol per initiating group) (Figure 1) and DMF (14.4 mL, 0.19 mol) was placed in a 25-mL round-bottom flask equipped with a magnetic stirrer, and the mixture was stirred until the powder was completely dissolved. Then NaN_3_ (0.214 g, 3.3 mmol) was added to the flask and purged with argon for 30 min. The mixture was stirred on a magnetic stirrer at room temperature overnight. The reacted polymer was precipitated into methanol. The filtered powder was dried under vacuum at 50 °C.

### 2.5. Introduction of Distant Hydroxyl Groups to Polyimide 

To obtain PI4 initiator (Figure 8, see Result and Discussion section), an azide-alkyne cycloaddition was carried out between the azide groups of PI3 (Figure 8) and the acetylene groups of propargyl alcohol. The typical synthesis was as follows. A sample of PI3 macroinitiator (0.25 g, 0.32 mmol per initiating group) (Figure 8) was placed in a 25 mL Schlenk flask equipped with a magnetic stirrer. Propargyl alcohol (92.9 μL, 1.6 mmol) and PMDETA (66.7 μL, 0.32 mmol) were added, and then DMF (4 mL, 0.052 mol) was added using a syringe. The solvent and syringes were purged with argon. The flask was sealed with a rubber septum, and the mixture was stirred until the powder was completely dissolved. Then, three freeze−pump−thaw cycles (evacuation for 15 min) were carried out, after which the flask was filled with argon. After opening the septum, CuBr (0.0457 g, 0.32 mmol) was added to the reaction mixture in an argon flow, after which the flask was closed again with the septum, three more freeze−pump−thaw cycles (evacuation for 15 min) of the reaction mixture were carried out, the flask was filled with argon and thermostated in an oil bath, placed on a magnetic stirrer with a temperature regulator at 50 °C overnight. Then the reaction mixture was cooled to room temperature and the product was precipitated into methanol. Residual copper was disposed of by changing the precipitant and reprecipitation from DMF. The filtered powder was dried under vacuum at 50 °C.

### 2.6. Synthesis of Linear Homopolymer PCL with Terminal Alkyne Groups

The synthesis was carried out by ROP of CL in a solution in toluene using propargyl alcohol as an initiator. A typical synthesis procedure is as follows: Propargyl alcohol (51 μL, 0.89 mmol), ε-caprolactone (1.97 mL, 17.8 mmol), and toluene (2.52 mL, 23.7 mmol) were added to a 10 mL Schlenk flask equipped with a magnetic stirrer. Then three freeze−pump−thaw cycles (evacuation for 15 min) were carried out, after which the flask was filled with argon. Then, 0.08 mL (0.25 mmol) of Sn(Oct)_2_ was introduced in an argon flow into the reaction, after which the flask was closed again with a septum, three more freeze−pump−thaw cycles (evacuation for 15 min) of the reaction mixture were carried out, the flask was filled with argon and thermostated in the oil bath, placed on a magnetic stirrer with a temperature controller, at 100 °C for a specified time (3 h) and at 80 °C overnight. Upon completion of polymerization, the reaction mixture was cooled to room temperature and diluted with methylene chloride. The resulting solution was passed through a silica gel column to purify the product from catalyst and monomer impurities. The solution was then concentrated on a rotary evaporator and the product was precipitated into cooled petroleum ether. The polymer was dried under vacuum at 30 °C.

### 2.7. Synthesis of Grafted Copolyimides PI-g-PCL

To obtain the targeted copolymers, two approaches were used. The first approach consisted in ROP of CL on a macroinitiator with distant hydroxyl groups (“graft from”). A typical experiment was as follows. A 10 mL Schlenk flask equipped with a magnetic stirrer was charged with 0.05 g (0.054 mmol per initiating group) of PI4 macroinitiator (Figure 8), sealed with a rubber septum, and then 3 mL (26.9 mmol) of ε-caprolactone was introduced in an argon flow. The mixture was thermostated at 130 °C in an oil bath. After complete dissolution of the initiator, 0.123 mL (0.38 mmol) of Sn(Oct)_2_ was introduced into the flask in an argon flow, and the reaction mixture was thermostated for a preset time. The molar ratio of PI4/CL was 1/500. The amount of Sn(Oct)_2_ was 5 wt.% in relation to the monomer. Upon completion of polymerization, the reaction mixture was rapidly cooled to room temperature and diluted with methylene chloride. The resulting solution was passed through a silica gel column to purify the product from catalyst and monomer impurities. The solution was then concentrated using a rotary evaporator and the product was precipitated into cooled petroleum ether. The polymer was dried under vacuum at 30 °C.

The second approach was to carry out an azide-alkyne cycloaddition between the azide groups PI3 macroinitiator (Figure 8) and the alkyne groups of linear PCL. A typical reaction was carried out as follows. A weighed portion of PI3 macroinitiator (0.05 g, 0.064 mmol per initiating group) (Figure 8), PCL (0.447 g, 3.9 mmol per initiating group), and PMDETA (13 μL, 0.064 mmol) was placed in a 25-mL Schlenk flask equipped with a magnetic stirrer, then DMF (3.7 mL, 47.8 mmol) was added using a syringe. The solvent and syringes were purged with argon. The flask was sealed with a rubber septum, and the mixture was stirred until the powder was completely dissolved. Then, three freeze−pump−thaw cycles (evacuation for 15 min) were carried out, after which the flask was filled with argon. After opening the septum, CuBr (0.009 g, 0.064 mmol) was added to the reaction mixture in an argon flow, after which the flask was closed again with the septum, three more freeze−pump−thaw cycles (evacuation for 15 min) of the reaction mixture were carried out, the flask was filled with argon and thermostated in an oil bath placed on a magnetic stirrer with a temperature regulator, at 50 °C overnight. After that the reaction mixture was quickly cooled to room temperature and diluted two times with THF. To remove copper salts from the mixture, it was passed through a column filled with Al_2_O_3_, then concentrated using a rotary evaporator, and precipitated into cooled petroleum ether. The filtered powder was dried under vacuum at 30 °C.

### 2.8. Methods

^1^H NMR spectra were recorded on Bruker AC-400 (400.1 MHz), with the signals of the solvent (DMSO-d_6_, CDCl_3_) as the reference.

IR spectra were recorded using a Shimadzu IR Affinity-1S spectrophotometer in the ATR mode (multiple attenuated total internal reflection) with a 4 cm^−1^ resolution and 30 scans.

Molecular weights and dispersities (Ð) of the samples were estimated by size exclusion chromatography (SEC) on Agilent-1260 Infinity fitted with differential refractive index (RID), light scattering (LS), and viscometry (VS) detectors equipped with two Agilent PLgel MIXED-C columns (7.5 × 300 mm, 5 µm), 1 × 43 PLgel 5 M guard column (50 × 7.5 mm), and autosampler. The analysis was carried out at 50 °C using 0.1 M LiBr in DMF as eluent at a flow rate of 1.0 mL min^−1^. The salt was added to suppress the aggregation of macromolecules. For triple detection analysis the system was calibrated using PMMA standard with M_p_ = 2.16 × 10^3^ g mol^−1^ with dn/dc=0.052. Before analysis, all samples were passed through a 0.45 µm Nylon filters. SEC data were analyzed using Agilent GPC/SEC Software version 1.2.

Thermogravimetric analysis (TGA) of obtained copolymers was performed using a TG 209 F1 Libra analyzer (NETZSCH, Germany) in the temperature range of 25–800 °C and heating rate of 10 deg min^–1^ under nitrogen. The weight of samples was 2–3 mg. 

Melting temperatures and enthalpies of synthesized copolymers were determined by differential scanning calorimetry (DSC) using a DSC 204 F1 Phoenix device (NETZSCH, Germany) at a heating rate of 10 deg min^–1^ in the range from −30 to +120 °C under nitrogen. The weight of samples was 2.5–3.5 mg.

The wide-angle X-ray scattering (WAXS) was carried out on an upgraded DRON 2.0 diffractometer (Saint-Petersburg, Russia) with CuKα radiation with a 0.154 nm wavelength.

## 3. Results and Discussion

PCL blocks were introduced into copolyimides by two fundamentally different ways. In the first case, to obtain grafted copolyimides, a precursor molecular brush with poly(2-hydroxyethyl methacrylate) (PHEMA) side chains was synthesized. Hydroxyl groups located in each repeating unit of the side chains of such brushes were used as initiators for carrying out ROP of CL. As a result the "brush on brush" structures PI-*g*-(PHEMA-*g*-PCL) were obtained. The second approach was aimed at obtaining graft copolymers with PCL side chains directly attached to the PI backbone. For this, a combination of ROP and CuAAC was used in two different variations.

### 3.1. Synthesis of Multifunctional Polyimide Initiators

**Figure 2:**
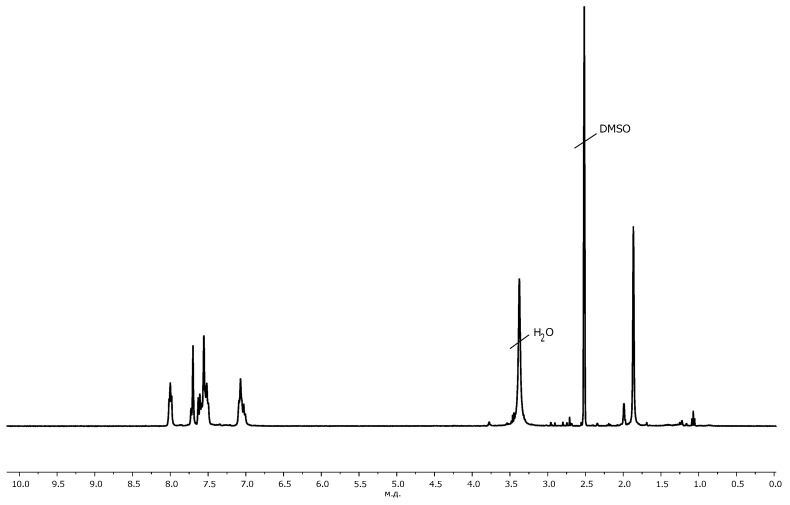
^1^H NMR spectrum of macroinitiator PI2.

The synthesis of multifunctional polyimide initiators with initiating OH-groups PI1 and 2-bromoisobutyrate groups PI2 in each repeating unit (Figure 1) based on soluble polyimide obtained by polycondensation of 4,4’-(1,3-phenylene-dioxy)bisphthalic anhydride and 2,4-diaminophenol was carried out according to a previously developed method [29]. The synthesis of polyimide was carried out according to the standard two-stage scheme with the preparation of polyamido acid (PAA) at the first stage and its subsequent thermal imidization in solution. During the synthesis of PAA, the equimolar ratio of monomers was strictly observed. The presence of a signal at 10.3 ppm in the ^1^H NMR spectrum of the product of thermal imidization of PAA indicated the presence of phenolic groups in PI1 (Figure 2). To obtain initiator PI2, samples of initiator PI1 were used as precursors, and treated with 2-bromoisobutyryl bromide under conditions that ensure almost complete esterification of the hydroxyl groups of PI1 [29]. The degree of functionalization by initiating 2-bromo-isobutyrate groups of PI2 initiator intended to perform ATRP, was 97 ÷ 99 mol% according to the ^1^H NMR spectroscopy data, i.e., virtually every repeating unit contained an initiating group.

**Figure 3:**
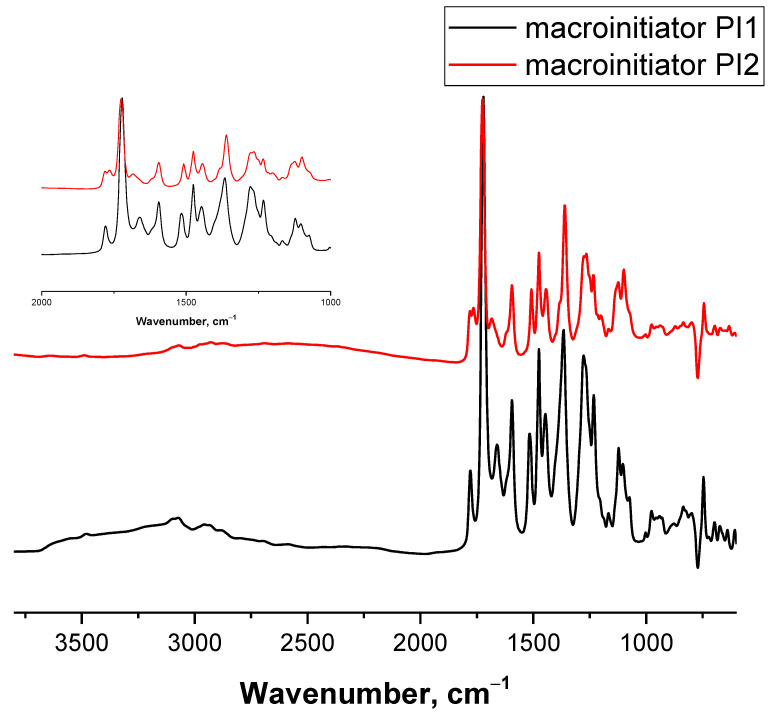
IR spectrum of macroinitiator PI1.

In the IR spectrum of PI1 (Figure 3), in addition to the characteristic bands of symmetric and antisymmetric vibrations of C=O groups of imide rings at 1780 and 1720 cm^−1^, respectively, there was an absorption band in the region of 1660 cm^−1^, which belongs to the vibrations of OH-phenol groups. The absence of the band at 1680 cm^−1^ indicates an almost complete conversion of *o*-carboxyamide groups of PAA to imide rings. It should also be noted that there are no absorption bands in the region of 1620 and 935 cm^−1^, the presence of which would indicate the development of benzoxazole rings during the high-temperature reaction with the participation of phenol and imide groups [31].

It should be noted that in an attempt to obtain initiator similar to PI1 but with a hydroxymethyl group in each repeating unit, during the thermal imidization of PAA based on (3,5-diaminophenyl)methyl alcohol an irreversible gelation rapidly develops at 170–180 °C in the N-MP solution which leads to a product insoluble in amide solvents. Apparently, interlink and/or interchain interactions of a more mobile and reactive (than phenol group) hydroxymethyl group leads to the formation of insoluble supramolecular structures due to hydrogen bonds.

### 3.2. Synthesis of Brush-on-Brush Type Copolyimides

**Figure 4:**
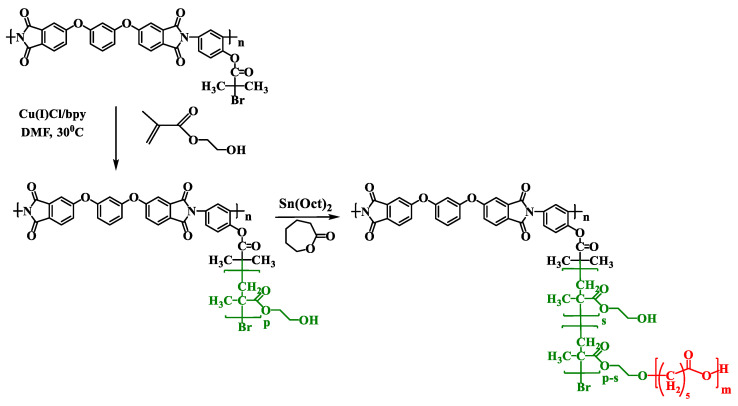
Synthesis of PI-*g*-PHEMA and PI-*g*-(PHEMA-*g*-PCL).

Previously, we reported [26], that polymerization of CL on PI macroinitiator PI2 (Figure 4) with phenol-type initiation groups practically does not proceed and the main polymerization product is homopolymer PCL. So, the introduction of PCL blocks into the side chains was carried out by performing ROP at the hydroxyethyl groups of the side chains of the polyimide brush with poly(2-hydroxyethyl methacrylate) side chains PI-*g*-PHEMA, which was used as a branched multifunctional macroinitiator. To obtain it, ATRP of 2-hydroxyethyl methacrylate was carried out on macroinitiator PI2. Table 1 shows the ATRP conditions and the monomer conversion values determined gravimetrically.

**Figure 5:**
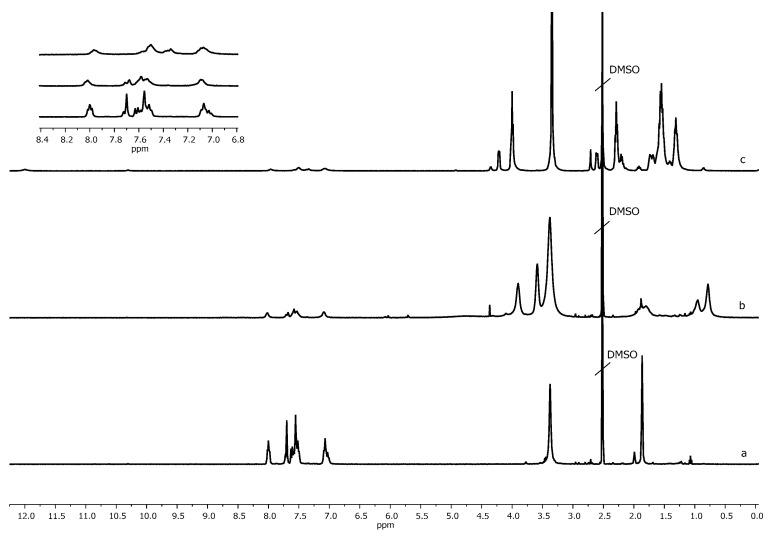
^1^H NMR spectra of macroinitiator PI2 (**a**), PI-*g*-PHEMA (**b**) and PI-*g*-(PHEMA-*g*-PCL) (**c**).

The formation of the copolymer PI-*g*-PHEMA was confirmed by the shift of the signals of the methyl protons of 2-bromo-isobutyrate groups from the 1.9 ppm to the 1.5 ppm in the ^1^H NMR spectrum of the ATRP products (Figure 5) and the appearance of signals of methylene protons of the –CH_2_–OH group at the 3.8 ppm while maintaining the signals of aromatic protons in the range of 6.0–8.5 ppm, related to the polyimide backbone. The intensities of the peaks from the PI backbone decreased due to its lower content in the final molecular brush.

**Figure 6:**
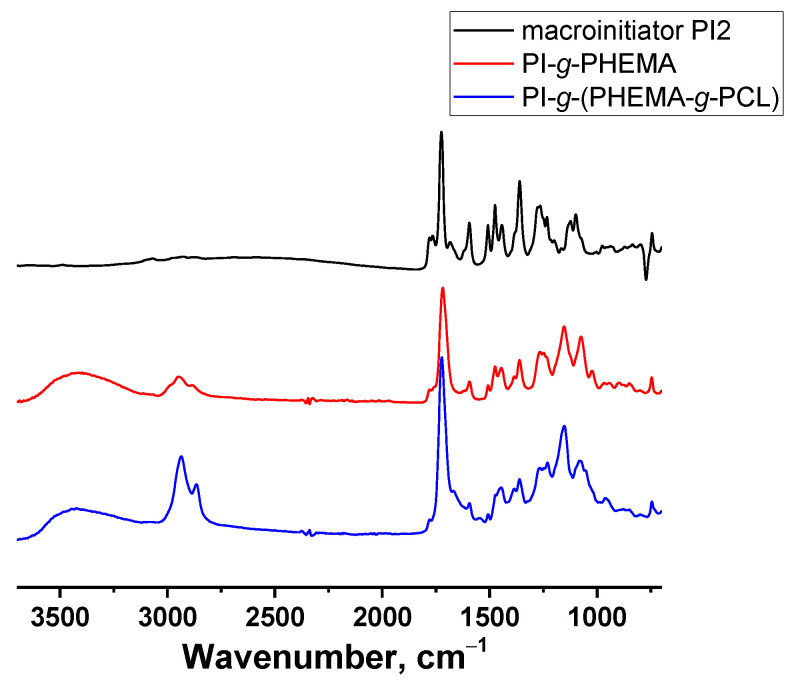
IR spectra of macroinitiator PI2, PI-*g*-PHEMA and PI-*g*-(PHEMA-*g*-PCL).

In the IR spectrum (Figure 6) of the HEMA polymerization product, in comparison with the spectrum of the macroinitiator, absorption bands of OH groups at 3500 cm^−1^, of methylene groups at 2950 cm^−1^ belonging to the PHEMA chains appeared, and the band at 1765 cm^−1^ disappeared, which refers to the vibrations of an ester group with an electronegative substituent (2-Br-substituent) in the α-position.

The structure of the obtained copolymers was proved by the complex use of ^1^H NMR spectroscopy and SEC. In the ^1^H NMR spectrum (Figure 5) of the CL polymerization product, signals of methylene protons –OCH_2_– and –COCH_2_– groups of PCL chains were observed at 4.1 and 2.3 ppm, respectively.

**Figure 7:**
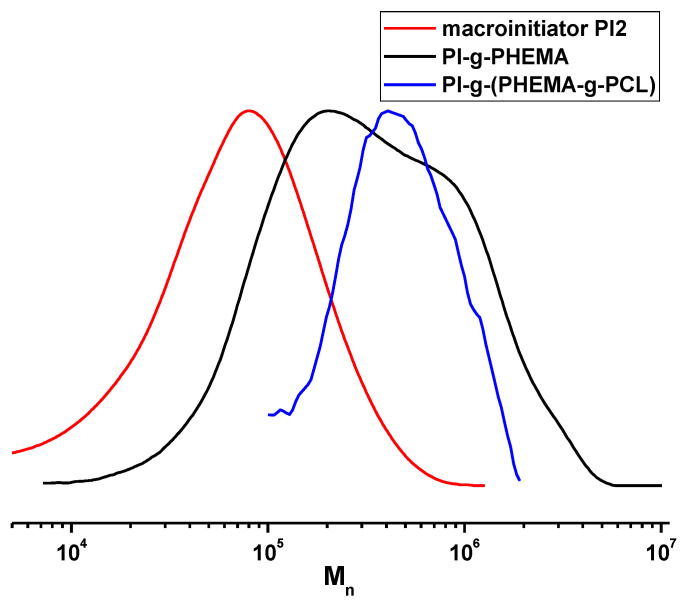
MWD curves of macroinitiator PI2, PI-*g*-PHEMA, and PI-*g*-(PHEMA-*g*-PCL).

Sequential offset of the MWD curves in the macroinitiator PI2, PI-*g*-PHEMA and PI-*g*-(PHEMA-*g*-PCL) series to the high MM values (Figure 7) and the presence of only one peak for PI-*g*-(PHEMA-*g*-PCL) indicates the grafting of PCL side chains to the PHEMA chains. According to the MW data corresponding to this series of polymers (Table 2), when passing from PI to the “brush on brush” structure, the MW increases by almost four times. A significant decrease in the value of the increment of the refractive index dn/dc in the series obviously reflects an increase in the ratio of aliphatic units in the copolymer. 

### 3.3. Synthesis of Molecular Brushes PI-g-PCL with a Polyimide (PI) Backbone and Polycaprolactone (PCL) Side Chains Using a Combination of ROP and CuAAC

**Figure 8:**
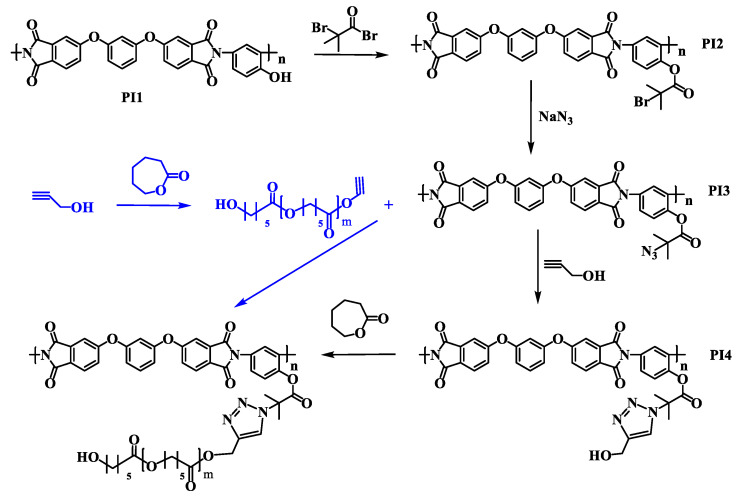
Routes for the synthesis of grafted copolyimides with PCL side chains by ROP and CuAAC.

To obtain the targeted copolymers PI-*g*-PCL, the following synthesis scheme was proposed, which includes two routes (Figure 8).

#### 3.3.1. Synthesis of PI with Azide Groups

**Figure 9:**
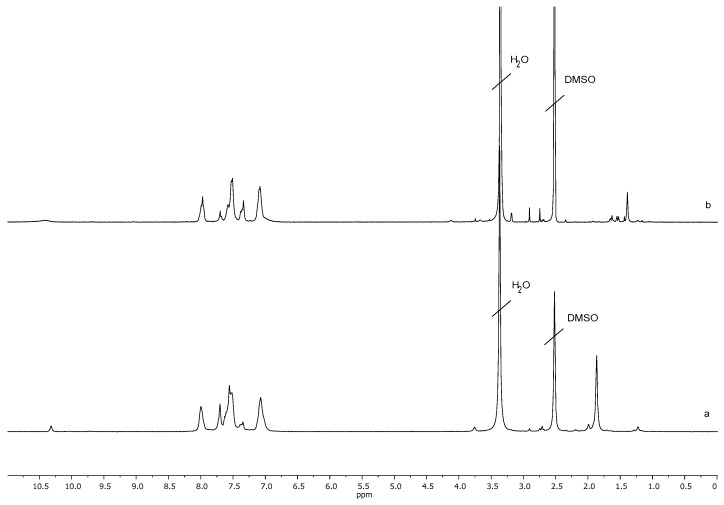
^1^H NMR spectra of macroinitiator PI2 (**a**) and macroinitiator PI3 (**b**).

At the first stage, samples of prepolymer PI3 (Figure 8) with azide functional groups were obtained. For this, polyimide macroinitiator PI2 (Figure 8) with 2-bromo-isobutyrate groups was modified under the action of sodium azide NaN_3_. Azidating agent was taken in 4 ÷ 10-fold excess. The reaction proceeded at room temperature for two hours. According to the ^1^H NMR spectra (Figure 9), functionalization with sodium azide proceeded completely, as a result of which the signal at 1.9 ppm shifted to the 1.35 ppm. The degree of functionalization was determined by the content of 2-bromo-isobutyrate groups in the initial polyimide macroinitiator, which was 60%.

Table 3 presents the molecular weight characteristics of the initial PI2 macroinitiator with 2-bromo-isobutyrate groups and the obtained PI3 macroinitiators with azide groups.

During the isolation of the azidation product, the polymer is apparently fractionated, which leads to a decrease in the dispersity and MW of the obtained macroinitiators PI3.

#### 3.3.2. Synthesis of PI with Hydroxyl Side Groups Attached to the PI Backbone through the 1,2,3-Triazole Linker

**Figure 10:**
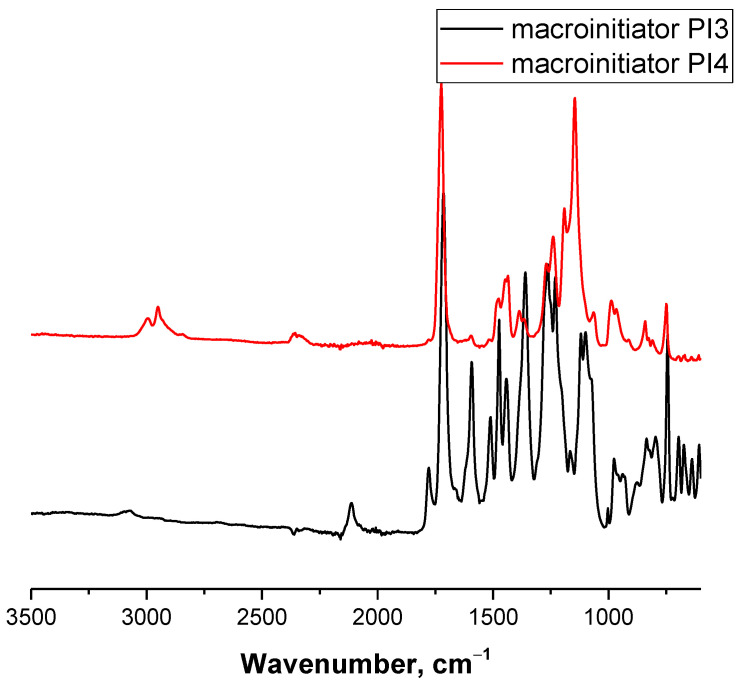
IR spectra of PI macroinitiators PI3 and PI4.

To implement the first route of the synthesis, the obtained prepolymer PI3 (Figure 8) was introduced into reaction with the propargyl alcohol. As a result of a click reaction, initiating ROP hydroxyl groups separated from the benzene ring of the backbone by a triazole ring (macroinitiator PI4, Figure 8) were introduced into the initiator. The reaction was carried out at a 50 °C for 2 hours in an N-MP using a 1.5-fold excess of propargyl alcohol and a CuCl/PMDETA catalytic system. Figure 10 shows the IR spectra of macroinitiators PI3 and PI4 (Figure 8). In the IR spectrum of the macroinitiator PI4, in addition to the characteristic absorption bands of imide rings (at 1370 cm^–1^ and a doublet at 1776 cm^–1^ and 1717 cm^–1^), there is a peak at 2300 cm^–1^, which refers to the stretching vibrations of the ethynyl group (–C≡CH) (Figure 10).

The resulting polymer PI4, according to the SEC data, had the following characteristics: *M_n_* = 34 × 10^−3^, *Đ* = 1.2.

#### 3.3.3. Synthesis of Molecular Brushes with a PI Backbone and PCL Side Chains by Polymerization of ε-Caprolactone on a Multicenter Macroinitiator PI4

**Figure 11:**
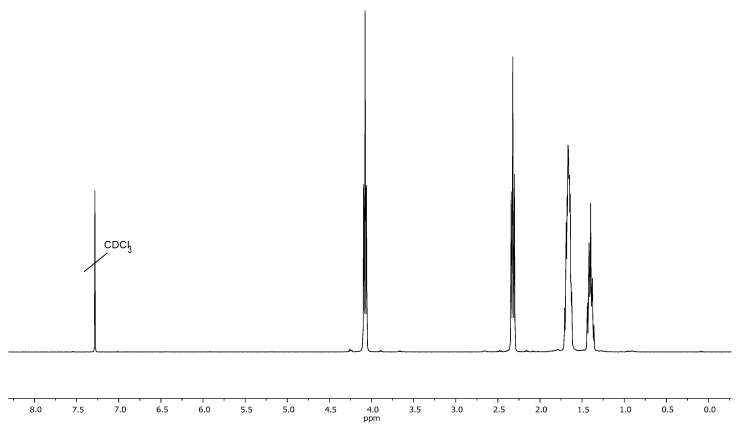
^1^H NMR spectrum of the polymerization product of CL on macroinitiator PI4.

The resulting macroinitiator PI4 (Figure 8) was used to carry out ROP of CL in bulk (initiator/monomer molar ratio = 1/1000) at 130 °C in an inert atmosphere in the presence of tin (II) octanoate as a catalyst. The ^1^H NMR spectrum of the obtained polymer (Figure 11) contains signals of methylene protons of the –OCH_2_– and –COCH_2_– groups at 4.0–4.1 ppm and 2.2–2.3 ppm and the middle methylene groups of the PCL side chains at 1.6–1.7 ppm and 1.35–1.45 ppm. In the spectrum of the polymerization product, signals of aromatic protons are not visible, due to the fact that in chloroform the polyimide is completely shielded by the PCL side chains.

Table 4 presents the polymerization conditions and MW characteristics of the obtained molecular brushes with PCL side chains.

**Figure 12:**
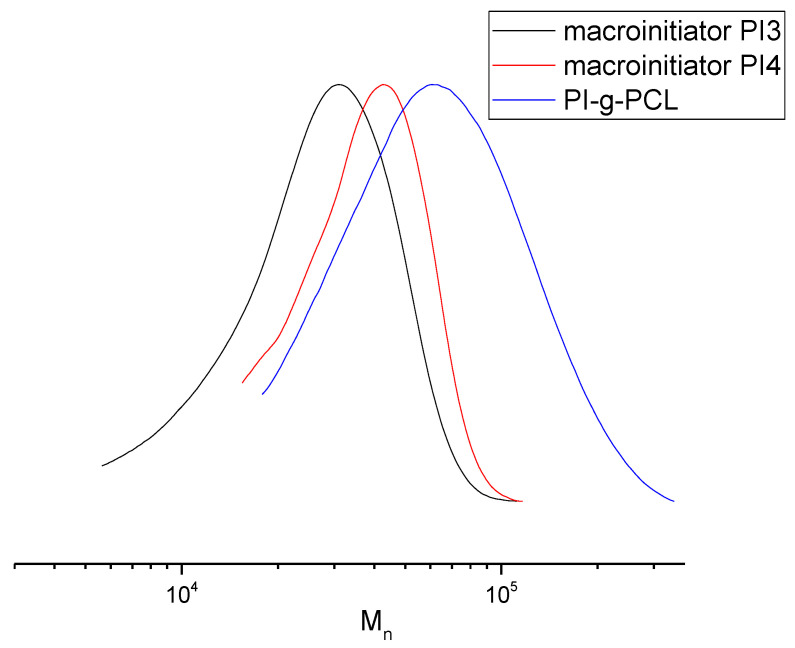
MWD curves of macroinitiators PI3 and PI4, and grafted copolyimide PI-*g*-PCL **5**.

Therefore, this approach allows to obtain graft copolymers with a PI backbone and relatively short PCL side chains, which is illustrated by the MWDs in Figure 12.

#### 3.3.4. Synthesis of Molecular Brushes with a PI Backbone and PCL Side Chains by CuAAC of Prepolymers

**Figure 13:**
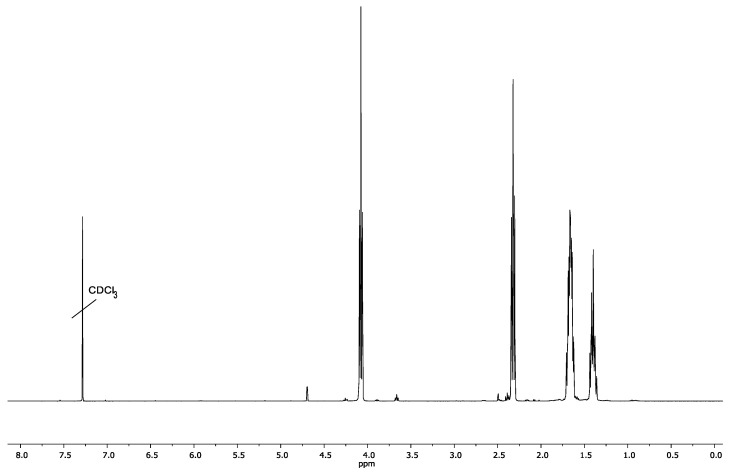
^1^H NMR spectrum of linear PCL with pendant –C≡CH groups.

To implement the second route, a linear PCL (Figure 8) with a terminal triple bond was synthesized. Polymerization was carried out in bulk at 100 °C using propargyl alcohol as an initiator and tin (II) octanoate as a catalyst. The ^1^H NMR spectrum of the polymerization product (Figure 13) contains all characteristic signals of PCL: signals of methylene protons of the –OCH_2_– and –COCH_2_– groups at the 4.0–4.1 ppm and 2.2–2.3 ppm and the middle methylene groups of the PCL chains at 1.6–1.7 ppm and 1.35–1.45 ppm. Moreover, clear signal at the 4.3 ppm, related to the protons of the –C≡CH end groups is visible in the ^1^H NMR spectrum (Figure 13).

Table 5 presents the polymerization conditions and MW characteristics of the obtained linear PCL with the –C≡CH end groups.

Azide-alkyne cycloaddition was carried out between linear PCL and macroinitiator PI3 (Figure 8), as a result of which the targeted copolyimides PI-*g*-PCL were also obtained. The click-reaction was carried out in an N–MP at 70 °C for 24 h, using CuCl/PMDETA as the catalytic complex. The molar ratio of the reagents was PI3/PCL/CuCl/PMDETA = 1/2/1/1.

Table 6 presents the MW characteristics of the products of click-reaction between linear PCL and macroinitiator with azide groups PI3. It can be seen that the implementation of the second approach leads to a more significant increase in molecular weight (Table 6), but unreacted linear PCL remains in the system. It is found in the form of a second peak in the chromatogram in the lower molecular weight region and its MW completely coincides with the mass of the previously analyzed linear PCL with alkyne end groups.

### 3.4. TGA, DSC and X-ray Study of the Synthesized Copolymers

**Figure 14:**
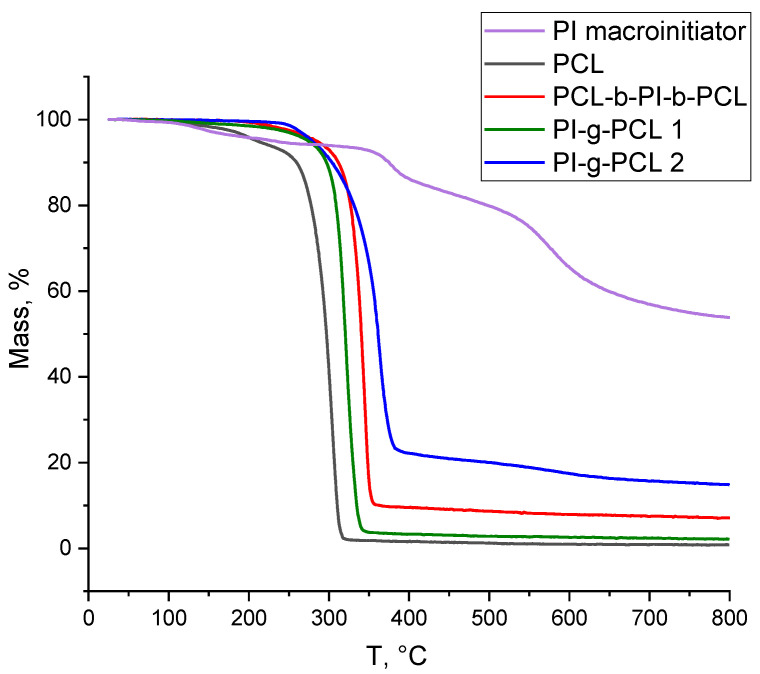
TGA curves for the synthesized macroinitiators, macromonomer, and copolymers.

Table 7 and Figure 14 presents the data on the thermal stability of the PI macroinitiator, PCL macromonomer, and linear and grafted copolyimides based on it. All copolymers lost most of the weight before 400 °C. But they are mostly stable till 275 °C, while linear PCL start to decompose already at 210 °C. The residual mass depends on the content of PI block in the copolymer. While linear PCL retains less than 1% of mass after 800 °C, molecular brush with long PCL side chains (PI-*g*-PCL 1)–2%, and molecular brush with short PCL side chains (PI-*g*-PCL 2)–15%.

**Figure 15:**
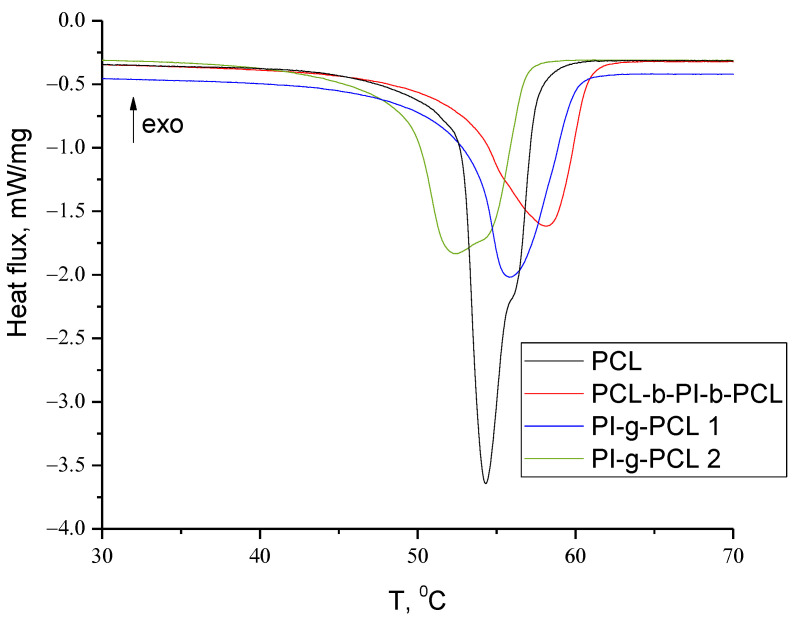
DSC curves for the synthesized macromonomer and copolymers.

Melting temperatures (T_m_) and enthalpy (ΔH_m_) of copolymers determined by the DSC method are presented in Table 7. The DSC curves of synthesized copolymers are presented with the endothermic melting peaks (Figure 15). All copolymers melt in the range from 52 to 58 °C and the melting enthalpy increases with the increase of PI content in the copolymer.

**Figure 16:**
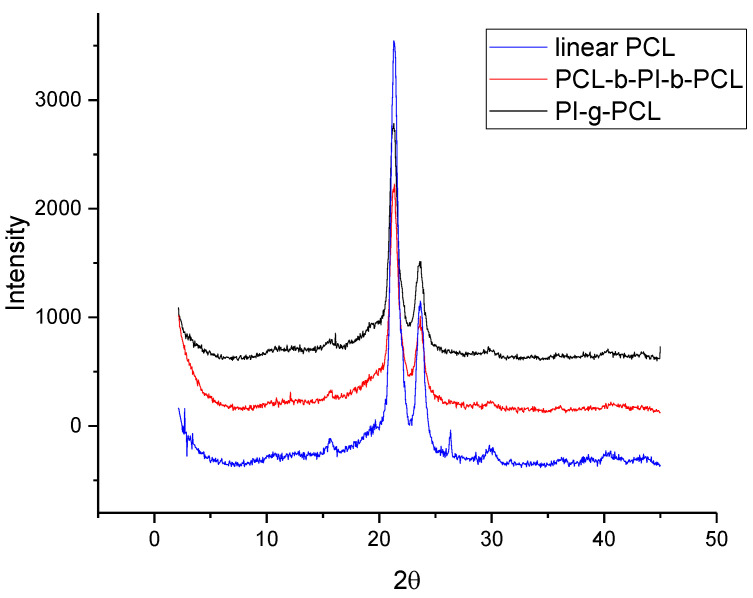
Diffractograms of linear PCL, triblock-copolymer PCL-*b*-PI-*b*-PCL, and grafted copolyimide PI-*g*-PCL.

Comparison of diffractograms of linear PCL with films of its linear and grafted copolymers showed that they are practically symbate (Figure 16). Consequently, the introduction of a polyimide block into the polycaprolactone matrix practically did not lead to a change in the degree of crystallinity of PCL. Apparently, the length of the PI blocks between PCL blocks is not enough for the PI to form the amorphous areas in the copolymer, so they’re ordered inside PCL crystalline structure. 

## 4. Conclusions

Multicomponent copolymers, in which PI blocks are combined with blocks of biocompatible aliphatic polymers, have great potential for the development of materials for tissue engineering. The introduction of PCL blocks into multiblock copolymers is of considerable interest from the point of view of further applications of these copolymers since PCL blocks are capable of undergoing alkaline and plasma etching and biodegradation. The synthesis of new initiators and the ROP of monomers and macromonomers containing functional groups on such initiators is a promising strategy for the preparation of macromolecules of complex architecture.

In this work we suggested an approach to the synthesis of the novel polymer molecular brushes with a polyimide (PI) backbone and poly(ε-caprolactone) (PCL) side chains. Using combination of polycondensation, ATRP, ROP, and CuAAC – targeted grafted copolyimides PI-*g*-PCL were synthesized as well as more complex “brush on brush” structures PI-*g*-(PHEMA-*g*-PCL). Comparison of different combinations of ROP and CuAAC showed that polymer-analogous transformations of a multicenter PI macroinitiator with an initiating hydroxyl group separated from the main chain by a triazole ring and carrying out ROP on it allows obtaining graft copolymers with a PI backbone and relatively shorter PCL side chains. While a separate synthesis of macromonomers with the desirable functional groups (polyimide with azide groups and PCL with terminal alkyne groups), followed by a click reaction leads to a more significant increase in molecular weight, but unreacted linear PCL remains in the system. X-ray scattering data showed that the introduction of a polyimide block into the polycaprolactone matrix practically did not change the degree of crystallinity of PCL.

## Figures and Tables

**Table 1:** Synthesis of PI-*g*-PHEMA by ATRP on macroinitiator PI2 in DMF solution.

NO	PI2/bpy/CuCl/HEMA *	[HEMA],%	T, °C	t, h	α, %
1	1/3/1/50	12	30	1	13.3
2	1/3/1/50	12	30	2	19.4
3	1/3/1/50	12	30	20	gel

*–molar ratio of the components in the reaction system, α–conversion of the monomer.

**Table 2:** MW characteristics of macroinitiator PI2, PI-*g*-PHEMA and PI-*g*-(PHEMA-*g*-PCL).

NO	Sample	*M*_n_ × 10^−3^	Ð	dn/dc, mL/g
1	PI2 macroinitiator	60	2.8	0.148
2	PI-*g*-PHEMA	180	3.9	0.087
3	PI-*g*-(PHEMA-*g*-PCL)	220	2.5	0.058

**Table 3:** MW characteristics of PI macroinitiators.

№	Sample	In/NaN_3_ Ratio	*M*_n_ × 10^−3^	*Ð*
1	Macroinitiator PI2	-	25	2.7
2	Macroinitiator PI3	1/10	22	1.3
3		1/5	17.5	1.2
4		1/5	20	1.2

**Table 4:** Polymerization conditions and MW characteristics of the PI-*g*-PCL.

№	In/M Ratio	t, h	*M*_n_ × 10^−3^	*Ð*
1	1/1000	1	43	1.7
2	1/500	2	52	1.5

**Table 5:** Polymerization conditions and MW characteristics of the linear PCL.

№	Solvent	In/M Ratio	*M*_n_ × 10^−3^	*Ð*
1	-	1/50	13.7	1.3
2	toluene	1/20	4.5	1.6

**Table 6:** MW characteristics of the click-reaction products.

Sample	Peak	*M*_n_ × 10^−3^	*Ð*
1	1	171	2.2
	2	13	1.5

**Table 7:** Thermal properties of synthesized copolymers.

Polymer	TGA			DSC	
	τ_5_ (°C)	τ_10_ (°C)	Mass Residua, %	T_m_, °C	∆H_m_, J/g
PI macroinitiator	232.9	375.0	53.83	-	-
PCL	210.5	259.6	0.82	54.3	−87.49
PCL-*b*-PI-*b*-PCL	284.6	311.7	7.08	58.1	−65.54
PI-*g*-PCL 1	274.3	296.9	2.12	55.8	−70.93
PI-*g*-PCL 2	279.1	303.7	14.81	52.3	−68.39

## Data Availability

The data presented in this study are available on request from the corresponding author.
